# Temporal visual representation elicits early auditory-like responses in hearing but not in deaf individuals

**DOI:** 10.1038/s41598-022-22224-x

**Published:** 2022-11-09

**Authors:** Monica Gori, Maria Bianca Amadeo, Francesco Pavani, Chiara Valzolgher, Claudio Campus

**Affiliations:** 1 Unit for Visually Impaired People, Fondazione Istituto Italiano di Tecnologia, Via Enrico Melen 83, 16152 Genoa, Italy; 2 Center for Mind/Brain Sciences (CIMeC), University of Trento, Trento, Italy; 3 Centro Interateneo di Ricerca Cognizione, Linguaggio e Sordità (CIRCLeS), University of Trento, Trento, Italy; 4 Integrative, Multisensory, Perception, Action and Cognition Team (IMPACT), Centre de Recherche en Neuroscience de Lyon (CRNL), Bron, France

**Keywords:** Sensory processing, Auditory system, Visual system

## Abstract

It is evident that the brain is capable of large-scale reorganization following sensory deprivation, but the extent of such reorganization is to date, not clear. The auditory modality is the most accurate to represent temporal information, and deafness is an ideal clinical condition to study the reorganization of temporal representation when the audio signal is not available. Here we show that hearing, but not deaf individuals, show a strong ERP response to visual stimuli in temporal areas during a time-bisection task. This ERP response appears 50–90 ms after the flash and recalls some aspects of the N1 ERP component usually elicited by auditory stimuli. The same ERP is not evident for a visual space-bisection task, suggesting that the early recruitment of temporal cortex is specific for building a highly resolved temporal representation within the visual modality. These findings provide evidence that the lack of auditory input can interfere with typical development of complex visual temporal representations.

## Introduction

It is not clear to date which are the principles at the base of cortical organization in our brain. If we consider blindness for example, in some cases, visual regions of the brain reorganize being recruited by auditory and tactile sensory inputs e.g.^1–3^ . This cross-sensory recruitment has been associated with the improvement of some auditory and tactile skills of blind individuals. However, we have recently showed that this reorganization does not occur for the auditory space-bisection task, for which in sighted but not in blind individuals the visual cortex processes the auditory spatial signals^4^. A possible explanation for this result is that visual experience is crucial to develop some spatial properties and when it is not available the visual spatial cortical processing cannot properly develop.

Some previous studies supported a sensory-independent supramodal organization of the visual cortex (see^5,6^), suggesting that the supramodal principle might extend to other sensory regions. Although this kind of research is much more limited in deafness compared to blindness, several studies have shown sensory-independent task-selective recruitment of the auditory brain. For instance, the auditory language network mostly maintains its distinctive properties in the brain independently of the sensory modality being used as input. In deaf adults, researchers have repeatedly reported that the auditory regions typically recruited by spoken language processing, can be recruited during sign production e.g.^7,8^ and sign comprehension e.g.^9,10^. Apart from activations related to language, studies have only clearly documented task-selective recruitment in auditory cortices for the perception of visual rhythm^11^. Specifically, regardless of the sensory modality involved, perception of rhythms peaked in the same anatomic auditory regions—that is, the posterior and lateral parts of the high-level auditory cortex. Similarly, there is evidence that face processing recruits the cortical territory associated with voice processing (i.e., the temporal voice area, TVA) in early deaf^12^. Interestingly, other results showed that the large-scale topography of the auditory cortex does not differ between hearing and deaf individuals. Tonotopic-like large-scale functional connectivity patterns can emerge and be retained through life in prelingually deaf humans without auditory experience^13^. In addition, studies in deaf cats revealed that the auditory cortex mostly preserves anatomic connectivity patterns^14–16^. Similar to blindness, it follows that large-scale anatomic and functional connectivity patterns seem to be preserved following deafness in humans.

Therefore, as for the visual context, one might wonder to what extent auditory experience is necessary for the auditory brain to develop and function. Many behavioral studies agree that the auditory system is the most accurate sense to represent temporal information e.g.^17–20^. The benefits of training on audio temporal tasks have been shown to transfer unidirectionally to the visual modality^21^ and auditory trainings can modify visual rhythm perception^20^. In addition, psychophysical studies have revealed a strong audition’s role in time-bisection tasks, which require subjects to encode presentation timings of stimuli, keep them in mind, extract the relative time intervals between them, and compare estimates^22,23^. Similar to our studies involving space-bisection, we wondered whether recruitment of the auditory brain may be necessary for time-bisection regardless of the sensory modality being tested. Moreover, since this was the case^24^, our subsequent question was to what extent auditory experience is necessary for this neural circuit to develop. In hearing people, we demonstrated the existence of an early ERP response, compatible with activation of the auditory cortex, specifically elicited by the construction of complex temporal representation during a purely visual time-bisection task^24^. Here we hypothesised that if the auditory modality is fundamental for the creation of complex temporal representations then deaf participants should not be able to perform the same visual task, and the specific cortical activation observed in hearing people should be altered. In particular, we expected that a lack of audition should impact on the development of some visual temporal skills and the underlying neural circuits, limiting one’s ability to understand complex temporal relationships such as those involved in solving time-bisection tasks.

## Methods

### Participants

A group of 12 deaf (D) individuals (mean age ± SD: 40.8 ± 14.2 years old) and a group of 12 age-matched hearing (H) individuals (34.4 ± 11.5 years old, t(21.06) =  − 1.19, *p* = 0.2) were recruited to participate in this study. Clinical details of deaf participants are summarized in Table 1. All individuals reported normal vision and no history of neurological, cognitive or other sensory-motor deficits except for deafness. The research protocol was approved by the ethics committee of the local health service (Comitato Etico, ASL3 Genovese, Italy) and by the Ethical Committee at the University of Trento (protocol: 2016–025) and conducted in line with the Declaration of Helsinki. Participants provided written informed consent prior to testing. When requested, instructions were delivered using Italian Sign Language otherwise they were provided in written Italian. Deaf individuals were not allowed to wear hearing aid during the experiment. Data and/or code used in the study are available from the corresponding author upon direct request.

**Table 1:** Demographic information about deaf participants obtained through self-report questionnaires.

Participant	Age	Sex	Age at deafness detection	Hearing aid use	Age at sign language first exposure
S01	58	M	2–4 years old	Uses currently	6–7 years old
S02	39	F	4–7 years old	Uses currently	Never
S03	34	F	7 months old	Uses currently	19 years old
S04	36	F	1–2 years old	Uses currently	5–6 years old
S05	53	F	From birth	Used in the past	20 years old
S06	24	F	From birth	Used in the past	15 years old
S07	21	M	From birth	Uses currently	From birth
S08	40	F	From birth	Uses currently	4–5 years old
S09	43	M	2–4 years old	Uses currently	28 years old
S10	58	F	From birth	Uses currently	55 years old
S11	22	M	4–7 years old	Used in the past	13 years old
S12	61	M	4–7 years old	Used in the past	7–8 years old

### Stimuli and procedure

**Figure 1:**
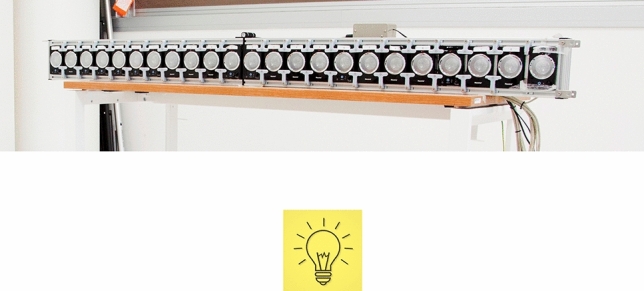
Experimental setup. Participants sat in a silent room, 180 cm away from the center of an array of 23 light-emitting devices spanning ± 25° of visual angle.

**Figure 2:**
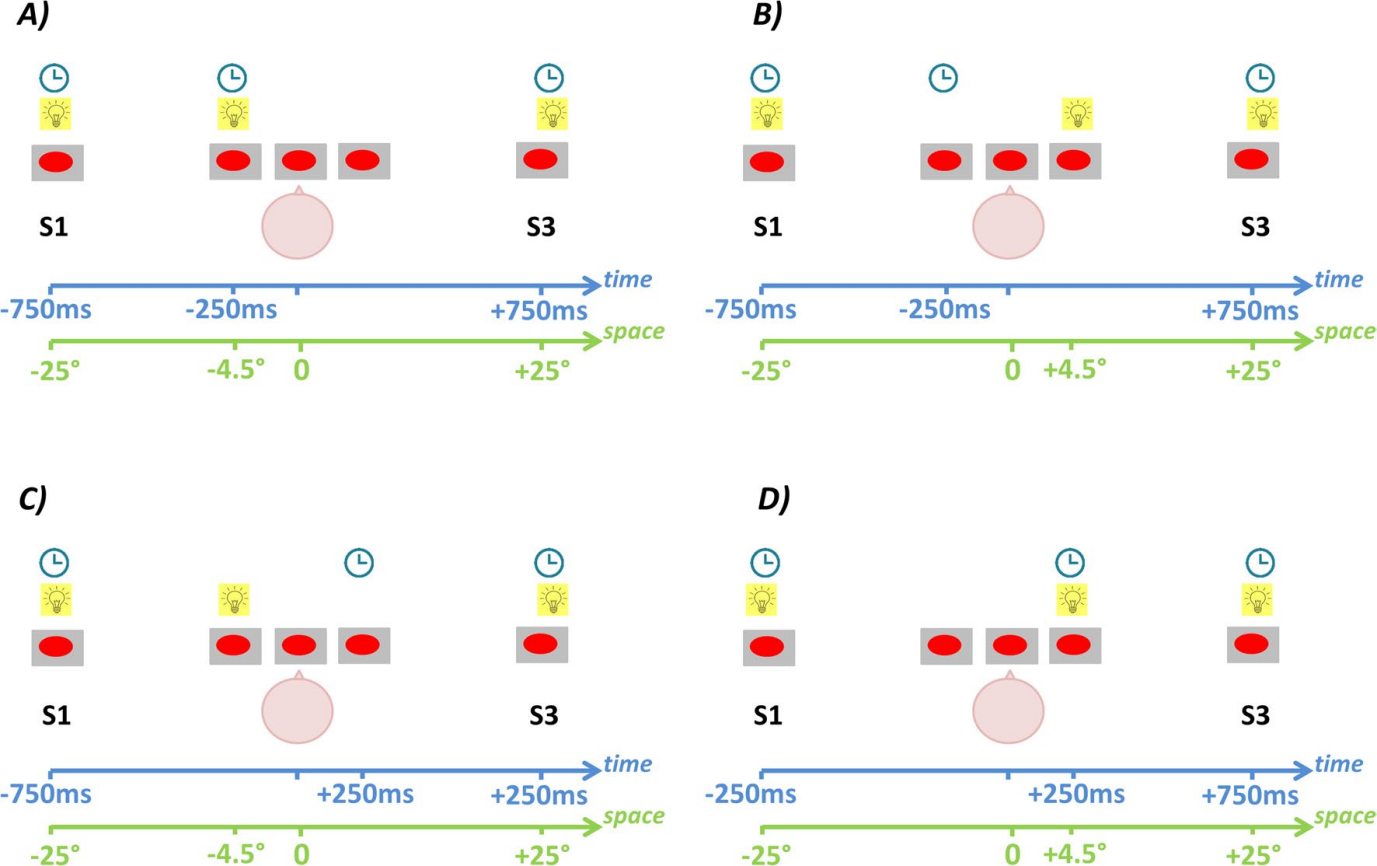
Experimental protocol for visual time- and space-bisection tasks. For each trial, individuals saw a series of three flashes (S1, S2, S3) that lasted 1.5 s with S1 and S3 at a fixed position of ± 25° with respect to the individual midline. S2 could occur randomly and independently at ± 250 ms (top and bottom panels) and from ± 4.5° (left and right panels) with regard to the physical spatial and temporal midpoints (0° and 0 ms). Interaction of spatial distance and temporal delay of S2 gave rise to four different conditions: (**A**) S2 from − 4.50° at − 250 ms; (**B**) S2 from + 4.50° at − 250 ms; (**C**) S2 from − 4.50° at + 250 ms; (**D**) S2 from + 4.50° at + 250 ms. S2 delivered from − 4.5°, and + 4.5° corresponds to S2 provided from the left or right side of the subject, respectively. Participants were instructed to judge whether the position of S2 in time or space (two separate blocks) was farther from S1 or S3 (bisection task).

**Figure 3:**
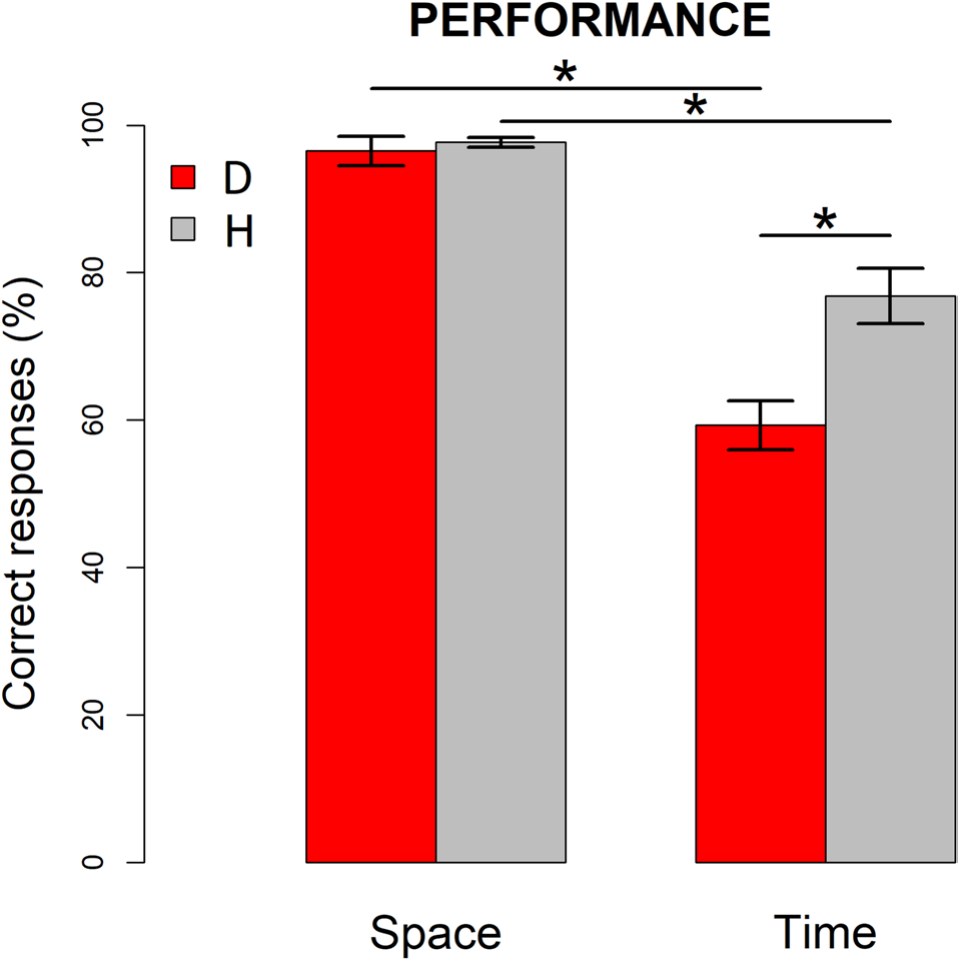
Performance (mean ± SEM) for spatial (left) and temporal (right) bisection tasks in deaf (red) and hearing (grey) subjects. Deaf participants show significantly lower percentage of correct responses compared to hearing participants in time- but not space-bisection. *p < 0.01 after Bonferroni correction.

**Figure 4:**
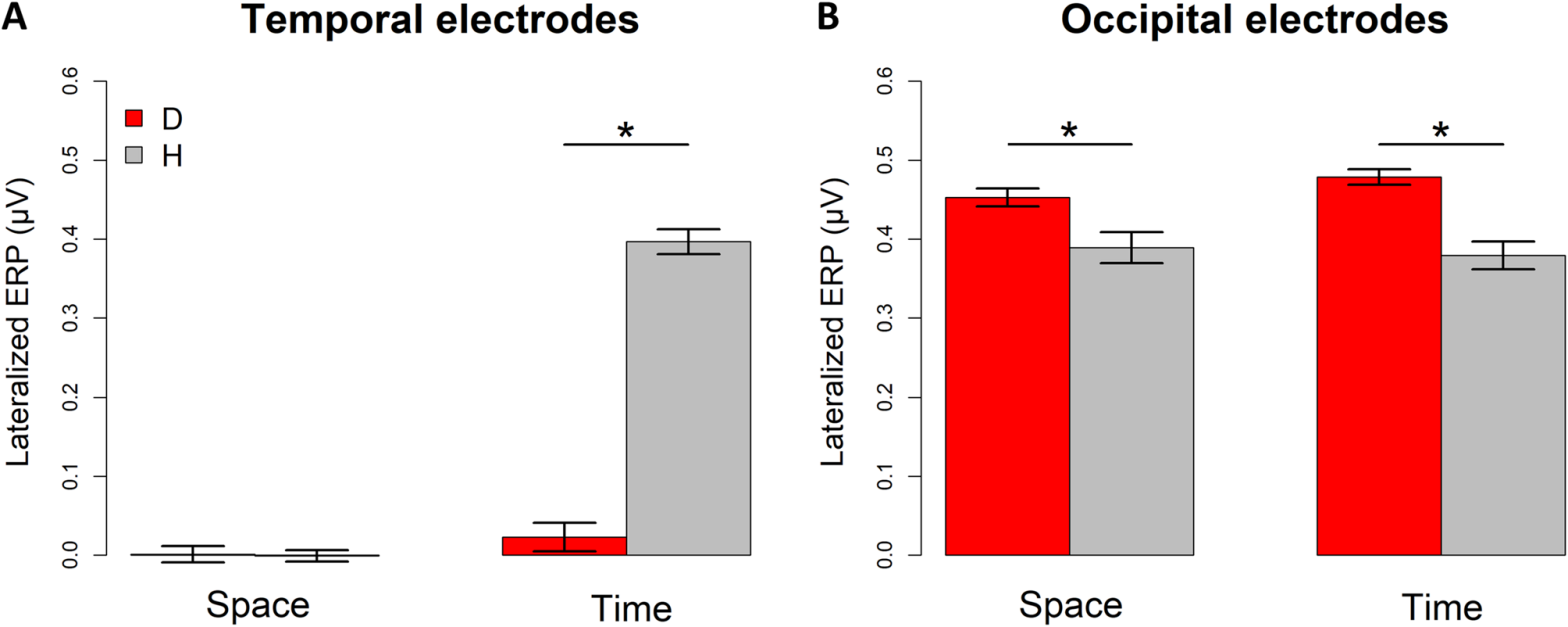
Lateralized (contralateral-minus-ipsilateral) ERP amplitude (mean ± SEM) in temporal and occipital scalp sites in the time window between 50–90 ms after the second flash of space and time bisection tasks. (**A**) temporal electrodes (T7/T8); (**B**) occipital electrodes (O1/O2). In grey, hearing participants; in red, deaf participants. The star indicates a significant difference between the groups (*p* < 0.001).

Participants sat in a silent room, 180 cm away from the center of an array of 23 light-emitting devices spanning ± 25° of visual angle (with 0° representing the central light-emitting device, negative values on the left, and positive values on the right; see Fig. 1). For each trial, three short flashes (namely S1, S2, S3; 2.3° diameter, 75 ms duration, and 20 cd/m^2^ luminance) were delivered at three different spatial positions and timings (Fig. 2). Participants performed a time- and a space-bisection task in two distinct separate blocks. The order of the two blocks was randomized across participants. Specifically, they judged whether S2 was temporally (time-bisection task) or spatially (space-bisection task) farther from S1 or S3. Stimuli were identical between blocks. The first (S1) and third flash (S3) were always delivered at − 25° and + 25° degrees respectively, with temporal separation fixed at 1.5 s. The second flash (S2) could occur randomly and independently from either − 4.50° or + 4.50° in space (Fig. 2 left and right panels), and at either − 250 ms or + 250 ms in time from the middle of the temporal flash sequence (Fig. 2 top and bottom panels). To avoid stereotypical responses, S2 was also presented at 0° and at 0 ms during catch trials (number of catch trials = 15). Each block consisted of 60 trials × 4 conditions: 1) S2 from − 4.50° at − 250 ms (i.e., S2 closer to S1 in space and in time; Fig. 2A), 2) S2 from − 4.50° at + 250 ms (i.e., S2 closer to S1 in space but closer to S3 in time; Fig. 2C), 3) S2 from + 4.50° at − 250 ms (i.e., S2 closer to S3 in space but closer to S1 in time; Fig. 2B), and 4) S2 from + 4.50° at + 250 ms (i.e., S2 closer to S3 in space and in time; Fig. 2D). Inter-trial interval was 1250 ± 250 ms. Previous experiments^24,25^ guaranteed that temporal separation between flashes was large enough to allow a complete decay of the ERP response. To avoid possible spurious neural responses, individuals were asked to answer using a pushbutton immediately after S3. We measured response times (i.e. the time between S3 and button press) in order to engage participants, and individual performance (i.e. the percentage of correct responses). Participants were warned to maintain a stable head position while fixating straight ahead. Their position, as well as their head orientation and EOG signal, were continuously monitored by the experimenters during the test. For more details about stimuli and procedure, see^24^.

### EEG data acquisition and pre-processing

A high-density EEG was recorded from 64 scalp electrodes using the Biosemi ActiveTwo EEG System. In order to monitor horizontal eye movements, two additional electrodes were placed at the left and right outer canthi for EOG recording. Thus, trials showing horizontal ocular movements were discarded by visual inspection.

EEG was filtered between 0.1 and 100 Hz. Transient high-amplitude artifacts from stereotypical (e.g. eye blinks) and non-stereotypical (e.g. movement, muscle bursts) events were removed using an automated artifact rejection method named Artifact Subspace Reconstruction (ASR), which is available as a plug-in for EEGLAB software^26,27^. ASR computed principal components which then spanned a lower dimensional subspace. Subspace components were compared to properties/results of decomposition from the baseline EEG (the algorithm identifies components from reference EEG data). From the component's activations, the root mean square amplitude is then estimated, as well as their mean and standard deviation. Given these statistics, a threshold matrix was calculated. The components derived during the processing were then compared to this threshold matrix to determine whether their variance had remained below the threshold. The reconstruction of data took place in the subspace. In this study, we used a sliding window of 500 ms and a threshold of 3 standard deviations to identify corrupted subspaces. The threshold was chosen to minimize the influence of occasional large-amplitude noise/artifacts, such as bursts originated from muscle contraction. Moreover, channels were removed if their correlation with other channels was inferior to 0.85, or if their line noise relative to signal was more than 4 standard deviations from the channel population mean. Time windows were removed when, after the application of the previously described criteria, the fraction of contaminated channels exceeded the threshold of 0.25. Other parameters were kept as default. EEG data were further cleaned using Independent Component Analysis^26^. Specifically, two EEGLAB toolboxes were used, namely SASICA^28^ and IC_MARC^29^, keeping all parameters as their default. For component rejection, criteria reported in the corresponding validation papers were followed, mainly based on abnormal topographies and/or spectra. The joint application of ASR and ICA allowed for obtaining a particularly good signal to noise ratio, and was complementarily efficient in removing two different kinds of artifacts. Given its application of sliding windows, ASR was especially efficient in removing transient artifact (e.g. short muscle contractions). Instead, ICA was applied to remove stereotyped repeated artifacts (e.g. cardiac or long-lasting muscle activities). After application of ASR, the runica function of the EEGLab toolbox automatically estimated the rank of the data and, when required, performed a preliminary dimensionality reduction with PCA, before extracting independent components. In addition, data were referenced to the average of all channels. For more details about EEG data acquisition and pre-processing, see^24^.

### Behavioral and sensor level analysis

Performance (i.e. percentages of correct responses) in the time- and space-bisection tasks were compared with two-way ANOVA, considering Group (H, D) as a between-subjects factor, and Task (Space, Time,) as a within-subjects factor. Post-hoc comparisons were conducted with two-tailed t-tests, with probabilities treated as significant when lower than 0.05 after Bonferroni correction.

The ERP analyses followed closely the procedures employed in a prior study investigating visual temporal abilities in hearing participants^24^, based on the hypothesis that deafness could lead to different early cortical responses during the time-bisection task (see^24^ for details). Specifically, we were interested in testing the hypothesis that hearing and deaf individuals show different early cortical responses after the second flash of the time-bisection task, which is considered the starting point for the construction of a temporal metric. We previously showed^24^ that in hearing people S2 during the time-bisection produces an activation in fronto-central and contralateral temporal areas, likely mimicking what is observed in auditory tasks. Thus, we focused our analyses on the neural responses to S1 and S2. While S2 can be considered the starting point for the development of a temporal or spatial metric, S1 can be considered as a control. Neural responses to S3 were not taken into account since the last flash could involve more complex mechanisms related to the metric definition, and it could be compromised by behavioral answers. For the spatial and time-bisection tasks, EEG data were averaged in synchrony with S1 or S2 onsets to compute ERPs, considering a period of 200 ms before S1 onset as a baseline for both flashes. After cleaning procedures, the total number of trials was around 1570 for each condition, approximately 54 per subject. Conditions were subsequently merged based on S2 spatial position ensuring approximately 108 trials per subject for each cell of the experimental design. Conditions were merged based on S2 spatial position without considering the temporal coordinates of S2 as we found no differences between the S2 responses when S2 was presented at − 250 ms or + 250 ms.

Based on our hypothesis and on previous work^24^, we focused on electrodes linked to auditory (T7, T8) and visual (O1, O2) processing, and in a time window between 50 and 90 ms after each flash. The time window of interest has been selected during the same bisection tasks in hearing people using a timepoint-by-timepoint approach with Microstate EEGLab toolbox^30^. Mean ERP amplitude was computed for each group by averaging the voltage in the selected time window, merging conditions based on S2 spatial position (i.e. 120 trials with S2 delivered from − 4.5° and 120 trials with S2 delivered from + 4.5°). For both the bisection tasks, ERP waveforms were collapsed across conditions and the hemisphere of recording (left, right) to obtain ERPs recorded on the contralateral hemisphere and on the ipsilateral hemisphere with respect to stimulus characteristics in space. Then, lateralized ERP responses were calculated as the relative difference between the contralateral and ipsilateral responses.

To investigate the differences between groups, the mean lateralized ERP amplitudes in the selected time window were analyzed in an omnibus ANOVA considering as factors Flash (S1, S2), Task (Space, Time), and Group (H, D). Two different ANOVA were performed, one considering the auditory (T7 and T8) and one considering the visual (O1 and O2) neural responses. ANOVA and two-tailed t-tests were conducted as post-hoc comparisons with probabilities treated as significant when lower than 0.05 after Bonferroni correction, applied to each subset of post-hoc comparisons separately. Moreover, in the previous study we showed that in hearing individuals there exists a strong correlation between ERP response in contralateral temporal sites and performance at the time-bisection task^24^. Here, we addressed the same association in deaf individuals using linear regression of mean lateralized ERP amplitude in the 50–90 ms time window against the percentage of correct responses. To further address the relationship between brain activity in temporal areas and performance, we realized ERP waveforms for temporal sites considering only correct trials.

Since acoustic areas are activated by visual lip reading in deaf people^7–10^, we checked the correlations between age at sign language first exposure and i) performance at the time-bisection task, ii) the lateralized ERP amplitude in temporal areas (50–90 ms time window).

### Source level analysis

We performed a distributed sources analysis using the Brainstorm software^31^ to investigate differences between hearing and deaf groups in the cortical generators of the ERP component taken into account. To get a more complete and understandable representation of sources, we did not consider the lateralized ERP, but the standard ERP responses. Then, cortical current source distribution within the brain was represented through 15,002 elementary dipoles obtained by sampling a tessellated cortical mesh template surface derived from the standard 1 mm resolution template of the Montreal Neurological Institute non-linear average of 152 subjects, processed with FreeSurfer 5.3 ICBM152;^32^. Since the individual MRIs were not available, the Brainstorm output, using a constrained approach, could be unrealistically precise (in terms of visualization). Therefore, to avoid misleading over-interpretation, dipole orientations were not normally fixed to the cortex surface but were unimpeded and able to assume whichever (unconstrained) orientation. The EEG forward modeling of volume currents was completed with a three-layered (head, outer, and inner skull) symmetric boundary element model (BEM) generated with OpenMEEG^33^. A diagonal noise covariance matrix was computed for each participant, using the pre-stimulus interval to estimate the sensor variance. The intensities of sources were estimated through the sLORETA approach^34^. This technique has been shown to be robust to noise in recorded data and to head model approximations with fair spatial resolution. In addition, the depth weighting used in this approach alleviates the natural bias of basic minimum norm estimation approaches toward superficial currents. Brainstorm’s default parameter settings have been used for both source reconstruction and BEM creation. We averaged source activation for each subject of the two groups and condition, within the selected time windows. Then, pairwise comparisons were investigated with a paired t-test, correcting results for multiple comparisons of source grid points with the FDR method^35^, using *p* = 0.0001 as a threshold. Based on our hypothesis, we were specifically interested in cortical generators evoked by S2 during the time-bisection task, and we compared the neural response to S2 between hearing and deaf individuals, considering the two tasks (spatial and temporal) and S2 positions in space (± 4.5°) separately. For more details about source level analysis see^24^.

### Ethics approval

The research protocol was approved by the ethics committee of the local health service (Comitato Etico, ASL3 Genovese, Italy) and by the Ethical Committee at the University of Trento (protocol: 2016-025) and conducted in line with the Declaration of Helsinki.

### Patient consent

Participants provided written informed consent prior to testing.

## Results

Twelve hearing and twelve deaf participants performed two visual bisection tasks. Subjects sat in front of an array of 23 LEDs, spanning ± 25° of visual angle (with 0° representing the central led, negative values on the left, and positive values on the right; see Fig. 1). They saw three flashes and judged whether the second flash was temporally (time-bisection task) or spatially (space-bisection task) farther from the first or the third flash. The first and third flash were always delivered at − 25° and + 25° degrees respectively, with temporal separation fixed at 1.5 s. The second flash was presented randomly and independently from either − 4.5° or + 4.5° in space, and at either − 250 ms or + 250 ms in time from the middle of the temporal flash sequence (see Fig. 2).

First of all, we demonstrated a behavioral difficulty of deaf participants in performing the time-bisection task (Fig. 3). The two-way ANOVA performed to investigate differences in the behavioral performance revealed a significant interaction (F(1,22) = 12.2, *p* = 0.002, generalized eta squared—GES = 0.2) between Group (H, D) and Task (Space, Time). Post-hoc t-tests revealed that performance of deaf individuals in time-bisection task (percentage of correct responses, mean ± SEM: 59 ± 3%) was significantly lower compared to both the temporal performance of hearing participants (percentage of correct responses: 76 ± 4%; t(21.7) = 3.48, *p* = 0.004;), and their own performance in the space-bisection (percentage of correct responses: 96 ± 2%; t(11) = 11.7, *p* < 0.001). Instead, no difference between groups was present in the space-bisection task, for which the probability of a correct response was comparable (t(13.4) = 0.55, *p* = 1).

Turning attention to the neurophysiological results, the omnibus ANOVA on the lateralized ERP amplitude involving temporal areas in the 50–90 ms time window showed a strong interaction between Flash (S1, S2), Task (Space, Time) and Group (H, D; F(1,22) = 193.3, *p* < 0.001, GES = 0.5). Thus, we subsequently performed hypothesis-driven follow-up ANOVAs and post hoc comparisons. First, we hypothesized that S2 could specifically modulate the interaction between other factors. Therefore, we performed two separate ANOVAs (one for each flash), with Task as within subject factors, and Group as between subject factor. As expected, we found a significant interaction between these two variables for S2 (F(1,22) = 224.9, *p* < 0.001, GES = 0.8). On the contrary, this was not the case for S1, where we did not find any significant effects (for the interaction: (F(1,22) = 2.4, *p* = 0.1, GES = 0.02). Thus, we focused subsequent analyses on S2, separately evaluating the two Tasks (Space, Time). We performed two separate ANOVAs (one for Space, the other for Time), with Group as between subject factor. We found a significant difference between Group for the time-bisection task (F(1,22) = 231, *p* < 0.001, GES = 0.9; Fig. 4A), but not for the spatial one (F(1,22) = 0.03, *p* = 0.9, GES = 0.001; see Fig. 4A). In the end, a post-hoc t-test revealed that S2 during the time-bisection task evoked a higher response in contralateral temporal areas of hearing compared to deaf people (t(45.2) =  − 15.6, *p* < 0.001).

**Figure 5:**
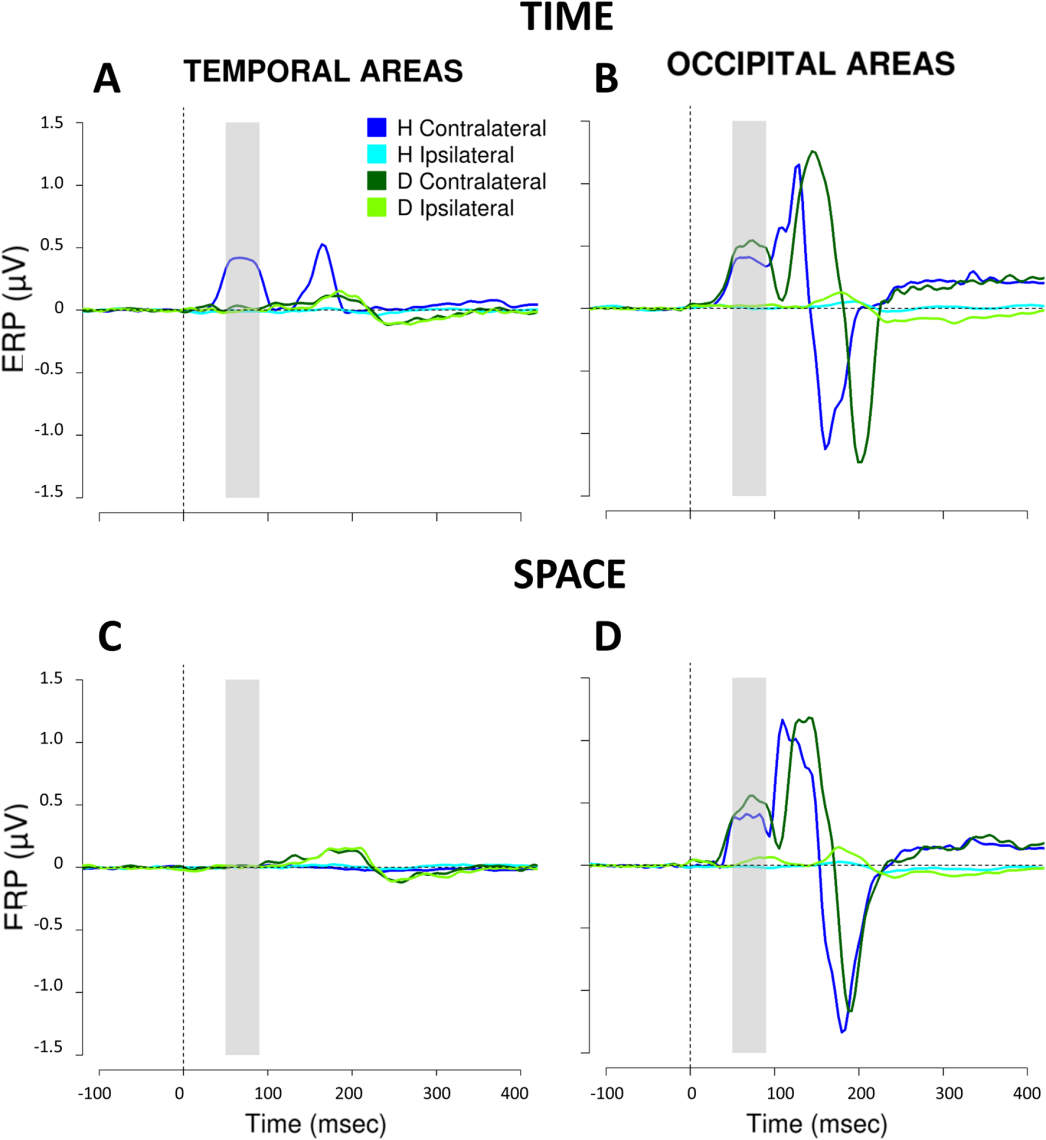
ERPs elicited by S2 during the time- (top) and space- (bottom) bisection tasks in temporal (left) and occipital (right) areas. ERPs collapsed over temporal (i.e., T7/T8)/occipital (i.e., O1/O2) scalp sites contralateral to the spatial side of S2 presentation are in dark blue and dark green for hearing and deaf groups respectively. ERPs collapsed over temporal/occipital scalp sites ipsilateral to the spatial side of S2 presentation are in cyan and light green for hearing and deaf groups respectively. On the x-axis, t = 0 is stimulus onset. The shaded area delimits the selected time window (50–90 ms).

**Figure 6:**
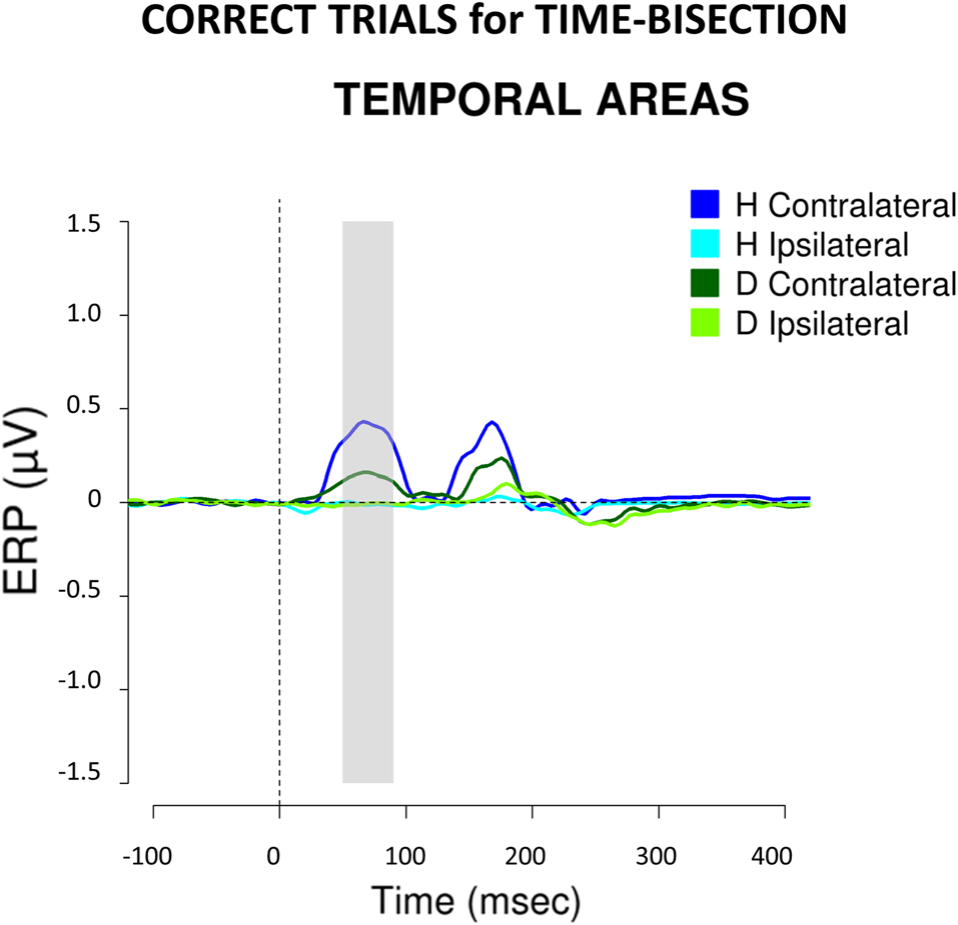
ERP elicited by S2 for the correct trials of the time-bisection task in temporal areas of deaf and hearing individuals. ERPs collapsed over temporal (i.e., T7/T8) scalp sites contralateral to the spatial side of S2 presentation are in dark blue and dark green for hearing and deaf groups respectively. ERPs collapsed over temporal/occipital scalp sites ipsilateral to the spatial side of S2 presentation are in cyan and light green for hearing and deaf groups respectively. On the x-axis, t = 0 is stimulus onset. The shaded area delimits the selected time window (50–90 ms).

This is evident in Fig. 5, representing ERP waveforms recorded over the temporal (Fig. 5, left panel) and occipital (Fig. 5, right panel) scalp contralateral and ipsilateral to S2, for time-bisection (top panel) and space-bisection tasks (bottom panel). Since waveforms for hearing people are identical to those described in^24^, here we focus on the differences between the two groups. Focusing first on the early time window of the time-bisection (Fig. 5, top panel), beyond the positivity in occipital areas, contralateral to stimulus position in space and evident in both groups, in hearing individuals S2 also elicits a strong positivity in contralateral temporal regions. This additional brain response is almost absent in deaf individuals. Thus, the response in temporal areas during the time-bisection task is specific for the hearing individuals. Moreover, this higher contralateral activation in temporal sites is associated with better performance in hearing people (r = 0.87, *p* < 0.001; in line with^24^) but this is not the case for deaf participants (r = 0.2, *p* = 0.6). In order to confirm the neural correlates underlying the performance at the time-bisection task, Fig. 6 shows ERP waveforms considering only correct trials for deaf and hearing individuals. It seems that, although weaker, a tiny activation in the early time window appears also in temporal areas of deaf participants.

As for occipital areas, an interaction between Flash (S1, S2), Task (Space, Time) and Group (H, D) also emerged from the omnibus ANOVA (F(1,22) = 14.8, *p* < 0.001, GES = 0.03). Hypothesis-driven follow-up ANOVAs revealed significant main effects of Group (F(1,22) = 290.3, *p* < 0.001, GES = 0.9 H: 0.37 ± 0.006 µV; D: 0.44 ± 0.005 µV) for S1, suggesting a higher activation for the deaf group. For S1, the main effect of Task (F(1,22) = 0.7, *p* = 0.4, GES = 0.02; Space, mean ± SEM: 0.41 ± 0.007 µV; Time: 0.41 ± 0.008 µV) and the interaction between Task and Group (F(1,22) = 3.7, *p* = 0.07, GES = 0.08) were not significant. For S2, the hypothesis-driven follow-up ANOVA showed a significant interaction between the two variables (F(1,22) = 6.8, *p* = 0.02, GES = 0.03; see Fig. 4B). Specifically, for S2, post-hoc t-tests showed a significant difference between groups for both the time- (t(15.1) = 4.11, *p* = 0.002) and the space- (t(13.1) = 3, *p* = 0.02) bisection tasks, while similar activation was present between the tasks within the hearing group (t(11) = 1.6, *p* = 0.3), and the deaf group (t(11) =  − 2.1, *p* = 0.1). A slightly bigger difference between the time- with respect to space-bisection task in deaf individuals is probably the cause of interaction between Task and Group for S2. To sum up, independently of the flash sequence, visual stimuli seem to elicit a higher response in occipital areas of deaf participants compared to hearing, and this occipital recruitment is even slightly higher for S2 during the time-bisection. This is evident in Fig. 5 (bottom panel) too. For both groups typical occipital ERP components are observed in the initial 200 ms following cue onset, but the amplitude of the early (50–90 ms) components is higher (Fig. 5B,D) for deaf individuals for both tasks (as previously reported by^36^).

The time window considered in the analyses was the first one presenting a task-related modulation (see Fig. 5). However, a later activation (P140) selective for the time-bisection task occurred in temporal areas of hearing and not deaf participants, and other latency differences emerged between the groups in occipital areas during time-bisection task.

The average response of the eye deviation measured by EOG did not significantly differ between the two groups (for time-bisection: t(11.3) =  − 1.19, *p* = 0.2; for space-bisection: t(11.4) =  − 1.02, *p* = 0.3). Moreover, as for hearing individuals (t(11) = 1.01, *p* = 0.3), within the deaf group there was no difference in eye deviation between the two tasks (t(11) = 1.38, *p* = 0.2).

**Figure 7:**
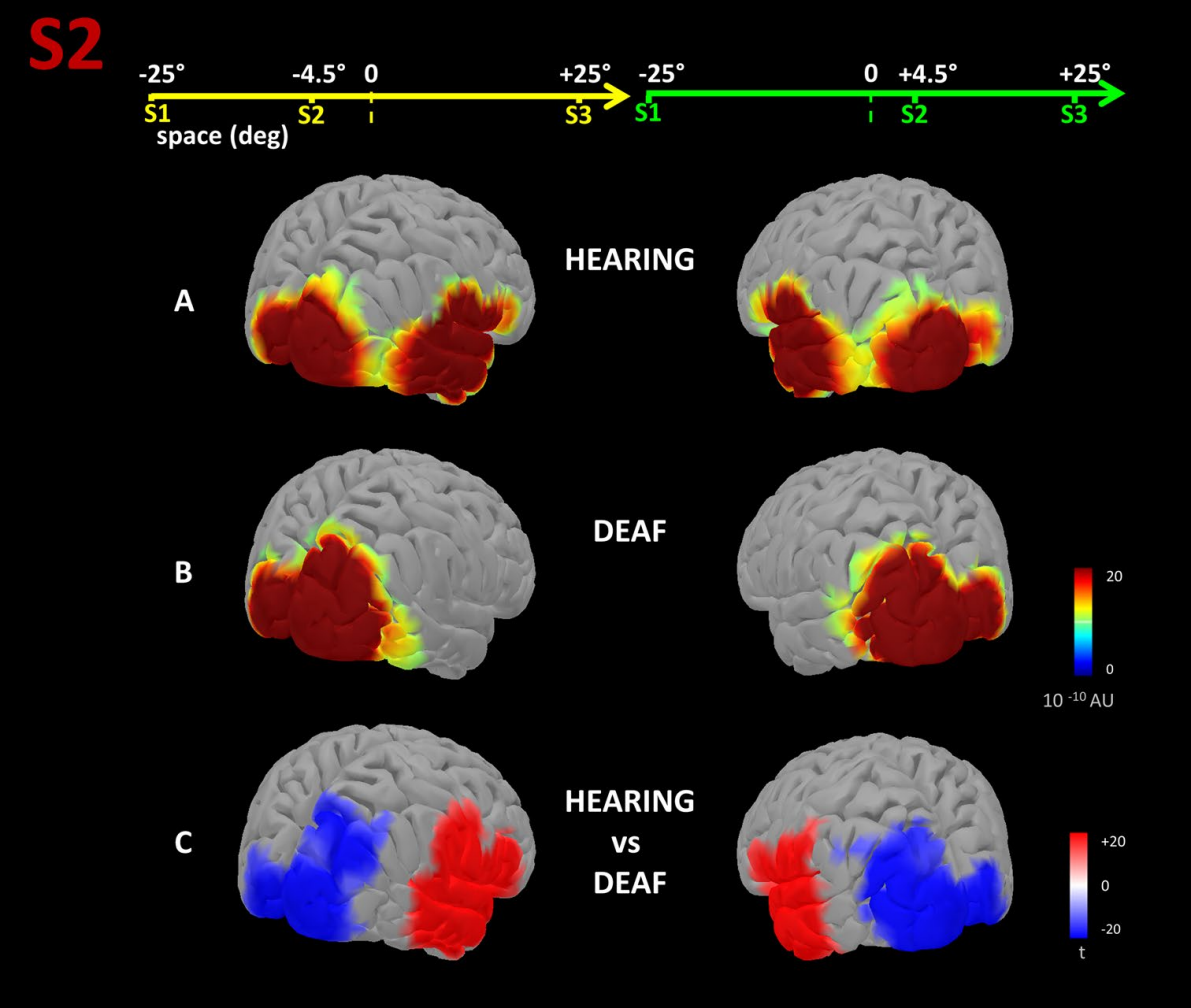
Average source activities within the 50**–**90 ms time window after S2 of the time-bisection task, are compared between hearing and deaf participants. Left and right panels report the conditions in which S2 was delivered from the left (i.e. − 4.5°) or the right (i.e. 4.5°), respectively. Average normalized source activation for hearing (**A**) and deaf (**B**) groups is reported in arbitrary (normalized) units (AU). Last line (**C**) reports the results of two-tailed t-tests; the scale is in terms of t-statistics. Significant values of t-statistics are displayed: reddish and bluish colors indicate stronger activations in time- and space-bisection tasks, respectively, while the intensity indicates the magnitude of t (i.e. the strength/significance of the difference). Only t-values corresponding to *p* < 0.0001 after FDR correction are displayed.

By comparing the groups at source level, we confirmed that the response of interest involves generators likely in the temporal cortices for hearing but not deaf individuals. Indeed, as evident in Fig. 7, S2 during the time-bisection task elicited a cortical response in the temporal region contralateral to the physical position of the stimulus in hearing and not in deaf people. The same experimental condition also evoked a response in the occipital region contralateral to the physical position of the stimulus for both groups, as expected for the processing of visual stimuli. However, in line with the statistical results involving the occipital electrodes, even the source analyses revealed that the recruitment of visual areas increases following deafness.

As regards the association between sign language exposure and time-bisection skills and neural correlates, we found correlations neither with the performance (r = 0.4, *p* = 0.3) nor with the early activation in lateralized temporal areas during the task (r = 0.02, *p* = 0.9).

## Discussion

Sensory modalities have a fundamental role in the development of specific cognitive skills. Nowadays, sensory cortices once assumed to process information of a specific sensory modality are known to code information deriving from different sensory channels^37^. This multisensory dimension of the sensory cortices has been referred to as supramodal, suggesting that the primary design principle underlying cortical architecture is task rather than the sensory modality^38^. Until now most evidence derives from studies about space processing in blindness (see^5^), with this work we add evidence to this theory from time processing in deafness. We have recently shown that in hearing people the auditory system has a role in processing complex temporal representations, even within the visual modality^24^. Here we show that the the auditory network involved in complex visual temporal representations develops in different manners in the lack of auditory experience.

An early activation compatible with a recruitment of the auditory cortex is observed in hearing people when processing visual flashes during a time-bisection task. In deaf participants, time-bisection of visual flashes does not elicit the same early (50–90 ms) responses in temporal cortices, for whose mostly the visual cortex is activated during the task. Specifically, we have previously observed that hearing individuals show, during time-bisection task, an early activation in fronto-central and contralateral temporal areas, which mimics some characteristics of the N1 ERP component usually peaking after the onset of auditory stimuli^39^. Similarities between the two components involve, for example, the early time window, the scalp areas, and the spatial selectivity. Indeed, the earliest subcomponent of the auditory-evoked N1peaks at around 70 ms, the component is mostly negative in front-central areas and inverts its polarity at mastoids^40^, and it is more pronounced in auditory areas contralateral with respect to the sensory input^39,41,42^. Based on these similarities, we suggested that the visual-evoked component in temporal areas of hearing individuals originates at the level of early sensory cortex, as the auditory-evoked N1 component, and requires similar mechanisms of early analysis elicited by auditory processing. The results of this work showing that deaf individuals are substantially less accurate when performing the temporal task and do not show similar neural activations suggest that the auditory input is necessary to develop a highly resolved temporal representations within the visual modality.

The difficulty in time-bisection following deafness was expected since the time-bisection consists of a complex high-order temporal representation task, which requires good memory and attention, and for whom the dominant role of audition has been previously demonstrated^22,23,43^. We exclude that it derived from impaired memory of the group of deaf individuals, since the two groups did not differ in their performance for the space-bisection task. The time-bisection difficulty we observed agrees with research that demonstrates the importance of auditory experience for the development of timing processing skills in other sensory channels ^44,45^. For example, both estimation of visual temporal durations in the range of seconds^46^ and tactile temporal durations in the range of milliseconds^47^ are compromised in deaf adults.

The main insight of the present study is the neural correlates of the deficit, which seem to correspond to the reduction of an early positivity in temporal sites contralateral to the stimulus position in space. The link between the early activation and time-bisection abilities is supported by the fact that in hearing people there is a strong association between the early activation in contralateral temporal cortices and percentage of corrected responses at the time-bisection task. The same association is lacking for deaf participants. Moreover, a similar, although weaker, early activation in contralateral temporal areas appears in deaf individuals when we analyzed their correct trials only. The fact that in deaf individuals the early response, likely involving the auditory cortices, is overall absent but it weakly appears when we considered correct trials could indicate that the activation in question may be actually the neural substrate underlying the performance, and auditory experience mediates its development. We can exclude that the difference between the two groups derives from eye-movements as the EOG analyses performed to evaluate eye deviations did not reveal significant differences between the two groups. Although the focus on the early component, it is worth mentioning that also a later component (P140) appeared to be modulated by the task in our study. Specifically, a P140 is selective for the time- and not space-bisection, and it occurred in temporal areas of hearing and not deaf participants. Future studies could investigate the origin and nature of this later component sensitive to temporal cues, whose emergence seems to be dependent on auditory stimulation too.

A recruitment of the auditory regions for visual and tactile inputs is often observed in deaf people e.g.^48–50^. As in blindness, also in deaf humans and deaf animal models this cortical recruitment has been associated with their behavior enhancement for the processing of visual stimuli in the periphery^51–53^. In this work, we investigated the central visual field of deaf individuals (+ 4.5° and − 4.5°). Activations of the auditory cortex for central visual stimuli has been shown for sign language comprehension^54–56^, face processing^12^, visual motion detection^57^ and detection of visual oddballs^48^. In a recent work, Bola et al.^11^, while suggesting a task-specific reorganization of the auditory brain, demonstrated that the auditory cortex of deaf people can sustain central visual perception too. They observed that visual rhythm processing involves posterior and lateral high-level auditory cortices, typically recruited during processing temporally complex sounds^58–61^. However, our study suggests that the cross-sensory neural reorganization in deaf individuals is not a general principle for the processing of visual temporal properties. We reveal the necessity of the auditory experience for the development of an early acoustic response selective for complex temporal properties of the visual stimuli during time-bisection tasks. Although rhythm and temporal information can be processed by different sensory modalities, audition processes such stimuli most efficiently (e.g.^62,63^). In line with this, our results demonstrate that, when audition is missing, the understanding of complex temporal relationships such as those involved in a time-bisection task and the underlying neural circuits are compromised.

Interestingly modifications are also observed on the visual cortex of deaf individuals. Indeed, independently of the flash sequence, visual stimuli seem to elicit a higher response in occipital areas of deaf participants compared to hearing. This result agrees with previous studies showing that independently of the task, deaf people show a higher activation in occipital areas compared to hearing participants^36,64^. Specifically, the activation is slightly more enhanced after the second flash of the time-bisection task, likely suggesting some attempts of compensation in the occipital brain for the lack of involvement of the temporal regions during the task. This is in line also with the study of Bolognini et al.^47^, suggesting that the recruitment of occipital areas following deafness is not always adaptive.

The impaired functional specialization observed in the auditory cortical areas of deaf individuals complements the results of the study we performed on the processing of auditory space-bisection in blindness. Complex spatial representation of auditory stimuli induces in sighted but not in blind people an early activation likely involving the visual cortex. In agreement with multisensory research showing a dominant role of vision in space perception (e.g.^65^) and audition in time perception (e.g.^18^), a speculation is that the visual cortices are involved in spatial processing and the auditory cortices are involved in temporal processing, all independent of sensory modality delivering the signal. Indeed, complex auditory spatial representation elicits specific activations in visual areas, while complex visual temporal representation elicits early activation in temporal regions. Although future researchers should test cortical activations that are involved in spatial and temporal representation in other senses, such as in the tactile modality or a multisensory context, our results strongly suggest that some domain-specific proprieties characterize the organization of the visual and auditory cortices^66^. Moreover, taken together, these findings add further evidence: consider there are some domain-specific aspects in the supramodal organization of sensory cortices, sensory experience could be a prerequisite for developing at least some of them. Indeed, lack of vision hampers neural correlates underlying some complex spatial abilities, and lack of audition hampers neural substrates of some complex temporal abilities. The existence of these early activations found in typical individuals and not in blind and deaf individuals suggests that multisensory neural connections are always present in individuals, but can be masked by the lack of typical sensory input and cannot be automatically recovered through plasticity.

To conclude, we observe that visual time-bisection elicits in hearing but not in deaf individuals an early response of the temporal cortex. Our results in typical hearing individuals suggest a supramodal organization of the auditory brain: audio-visual cortical interaction seems to occur at very early stages of processing and auditory regions could support complex visual temporal representations. Our results in deaf individuals add that this aspect of supramodal organization is dependent on sensory experience: the auditory experience seems crucial in developing an early temporal response specific for complex time perception of the visual stimuli. Besides shedding light on some limits of cortical reorganization following sensory deprivation, these findings offer important implications for understanding the neural underpinnings of temporal representations.

## Data Availability

Data and/or code used in the study are available from the corresponding author upon direct request.
